# Supplementary material for: The interactome of intact mitochondria by cross-linking mass spectrometry provides evidence for coexisting respiratory supercomplexes
Source: Mol Cell Proteomics. 2017 Dec 8;17(2):216–32. doi: 10.1074/mcp.RA117.000470 (PMC5795388; doi:10.1074/mcp.RA117.000470)
Supplement: Supplemental Data [file supp_RA117.000470_133922_0_supp_23978_fzffwf.zip › spectra_annotation/mito_DR_spectra_annotation/69-1-4-1-7-1.pdf]

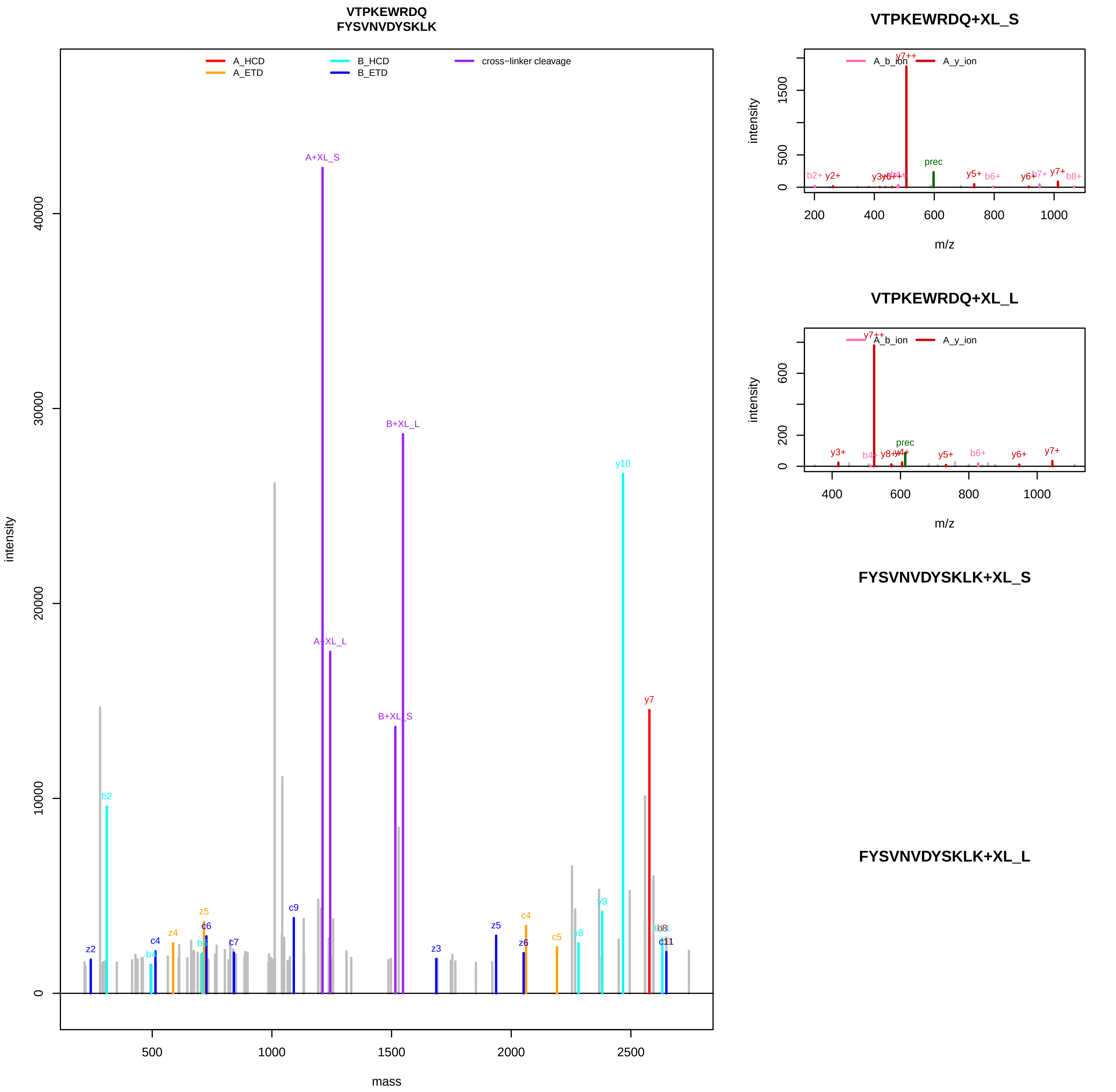

VTPKEWRDQ
FYSVNVDYSKLK
A_HCD
A_ETD
B_HCD
B_ETD
cross−linker cleavage
A+XL_S
B+XL_L
A+XL_L
B+XL_S
y10
y7
b2
z2
c4
b4
z4
z5
c6
b6
c7
c9
z3
z5
z6
c4
c5
y8
y9
c11
intensity
mass
0
10000
20000
30000
40000
500
1000
1500
2000
2500
VTPKEWRDQ+XL_S
A_b_ion
A_y_ion
y7++
prec
b2+
y2+
y5+
b6+
y6+
b7+
y7+
b8+
intensity
m/z
0
500
1500
200
400
600
800
1000
VTPKEWRDQ+XL_L
A_b_ion
A_y_ion
y7++
prec
y3+
b4+
y5+
b6+
y6+
y7+
intensity
m/z
0
200
600
400
600
800
1000
FYSVNVDYSKLK+XL_S
FYSVNVDYSKLK+XL_L
